# Cyrene-Enabled Green Electrospinning of Nanofibrous Graphene-Based Membranes for Water Desalination via Membrane Distillation

**DOI:** 10.1021/acssuschemeng.4c06363

**Published:** 2024-11-25

**Authors:** Antonios Keirouz, Francesco Galiano, Francesca Russo, Enrica Fontananova, Bernardo Castro-Dominguez, Alberto Figoli, Davide Mattia, Hannah S. Leese

**Affiliations:** † Department of Chemical Engineering, Faculty of Engineering and Design, University of Bath, Bath BA2 7AY, U.K.; ‡ Institute on Membrane Technology, National Research Council of Italy (CNR-ITM), Via Pietro Bucci 17/C, 87036 Arcavacata di Rende, Cosenza, Italy

**Keywords:** membranes, green solvents, PVDF, 2D materials, nanofibers, sustainable manufacturing, economic viability

## Abstract

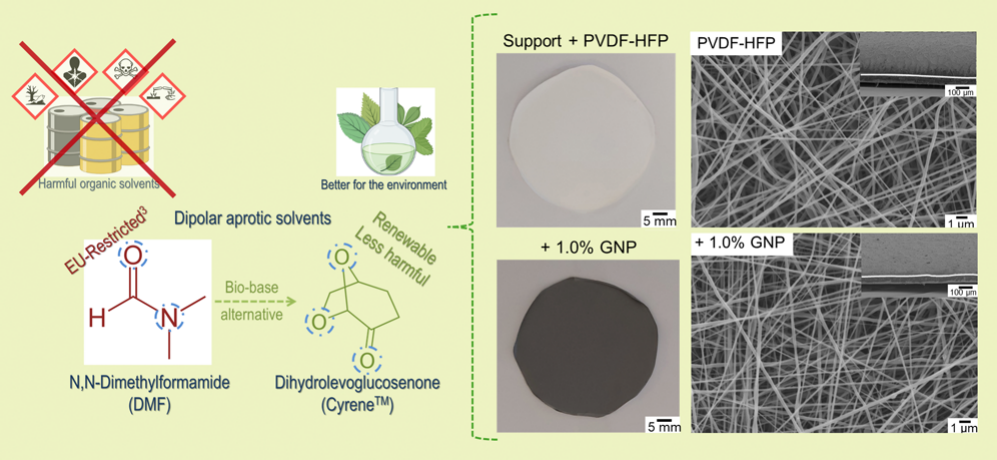

High-performance and sustainable membranes for water desalination applications are crucial to address the growing global demand for clean water. Concurrently, electrospinning has emerged as a versatile manufacturing method for fabricating nanofibrous membranes for membrane distillation. However, widespread adoption of electrospinning for processing water–insoluble polymers, such as fluoropolymers, is hindered by the reliance on hazardous organic solvents during production. Moreover, restrictions on industrial solvents are tightening as environmental regulations demand greener alternatives. This critical challenge is addressed here by demonstrating, for the first time, the fabrication of nanofibrous electrospun membranes of PVDF-HFP, poly(vinylidene fluoride)-co-hexafluoropropylene using a renewable, environment- and user-friendly solvent system containing Cyrene (dihydrolevoglucosenone), dimethyl sulfoxide, and dimethyl carbonate. The same solvent system was further used to produce nanocomposite graphene oxide (GO) and graphene nanoplatelet (GNP)-containing nanofibrous electrospun membranes. When tested for water desalination via membrane distillation, these membranes either outperformed or matched the performance of those produced with hazardous organic solvents, achieving salt rejection rates of >99.84% and long-term stability. The economic viability of the green solvent system was further validated through Monte Carlo simulations. This work demonstrates the potential to move fluoropolymer electrospinning from dimethylformamide-based systems to greener alternatives, enabling the consistent production of high-quality nanofibrous membranes. These findings pave the way for more sustainable manufacturing practices in membrane technology, specifically for water desalination via membrane distillation.

## Abstract 2

Cyrene-enabled electrospinning of water-insoluble polymers provides a vital alternative to toxic solvents and delivers high-performance nanofibrous membranes that meet or exceed the properties required for membrane distillation in water desalination, paving the way for high-performance and sustainable membrane manufacturing for water desalination applications.

## Introduction

The global demand for clean water is ever growing and membrane distillation (MD) is a promising alternative to water desalination in comparison to more conventional processes such as reverse osmosis which uses large amounts of energy.^1^ Although MD has proven to be an effective method for water desalination, the membrane fabrication process itself needs to be addressed, in particular, the use of toxic solvents to ensure the improved sustainability of the process,^1^ the importance of which has been highlighted in the recently published “12 principles of green membrane materials and processes”.^2^ The research in our paper exemplifies several of these principles including considering greener compounds, using less hazardous materials, and ensuring reproducibility and a robust performance.^2^ Electrospinning is a widely used technique for producing hydrophobic membranes for MD from continuous micro- and nanoscale fibers. However, the process remains heavily reliant on toxic (e.g., dimethylformamide) and halogenated (e.g., chloroform, trifluoroethanol) solvents.^3^ Several international bodies have introduced regulations to limit and ultimately refrain from the mass production and use of harmful solvents that harm the user and the environment. For instance, since December 2023, the Chemical Control Regulation in the European Union (REACH) and the UK have restricted the use of *N*,*N*-dimethylformamide (DMF), an aprotic solvent readily used in membrane fabrication, to minimize workplace exposure and environmental contamination risks.^4^ The USA has restricted the airborne exposure limit for DMF at 10 ppm averaged over an 8 h workday according to the National Institute for Occupational Safety and Health and the National Center for Environmental Assessment.^5^

Although green chemistry, as a concept, and the 12 Principles of Green Chemistry were established in the 1990s,^6−8^ research on electrospinning, until very recently, has neglected the investigation of green and sustainable solvents, with a negligible number of published articles focusing on processing water-insoluble polymers, such as fluorinated polymers and polyesters, via less harmful alternatives.^3,9^ A SCOPUS search utilizing the search term “TITLE-ABS-KEY electrospinning AND green AND solvent” reveals that only 0.5% of the total electrospinning literature specifically addresses green polymer processing. This estimate may be further reduced by a factor of 10, as many authors use the term “green” to refer to the processing of water–soluble polymers. Electrospinning needs the optimization of solution properties, processing parameters, and ambient conditions to yield electrospinnable solutions that can produce fibers with reproducible morphology.^10^ As electrospinning begins to overcome translational barriers, it is vital to address the substitution of toxic solvents with less harmful and preferably renewable alternatives for upscale manufacturing.

In addition to the use of green solvents, there is also a growing need to identify more sustainable alternatives to fossil fuel-derived polymers. However, many of these, e.g., poly(vinylidene fluoride) (PVDF), remain in heavy use, due to their unique, resilient properties, including as membranes for water treatment and desalination.^11^ There is no question that alternatives for fluoro-based polymers are also required, but in tandem, while these materials are still in heavy use industrially, it is vital that more sustainable ways of processing them are employed. As such, while looking for an alternative, minimizing the environmental impact of their current manufacturing is a necessary pursuit. The large surface-area-to-volume ratio of electrospun membranes further increases the topographical features of PVDF membranes, improving the flux, lowering the required operating pressure, and providing good retention of multivalent anion salts, high rejection rates, and chemical stability while keeping manufacturing costs low.^12,13^ The processing of PVDF into membranes using green solvent alternatives via methods, such as drop-casting and leaching, has been previously reported,^14−17^ but this has not been achieved, so far, via electrospinning. Electrospun nanofibers based on PVDF have been readily produced with conventional solvents such as DMF.^18^

Membrane distillation (MD) is an expanding technology which requires high-performance membranes—featuring high hydrophobicity, high permeance, low fouling, low thermal conductivity, high liquid entry pressure, and excellent mechanical strength—for large-scale applications.^19^ Due to their properties, 2D materials, including graphene derivatives such as functionalized graphene oxide (GO), reduced graphene oxide (rGO), and graphene nanoplatelets (GNPs), have been utilized as additives to produce composite membranes and their performance in MD composite membranes has been encouraging. However, to the authors’ knowledge, the electrospinning methodologies utilizing PVDF to produce graphene-based composite polymeric membranes to date have only used toxic, amide-based solvents including *n*-methyl-2-pyrrolidone (NMP),^20^ DMF,^21−23^ and DMAc.^24,25^ These nanofibrous electrospun graphene-based composite membranes have been used in membrane distillation for water desalination^26^ and for the removal of environmental pollutants (e.g., arsenic).^27^

Dihydrolevoglucosenone (commercially, Cyrene)^28,29^ is a biorenewable dipolar aprotic solvent alternative to NMP, DMF, and dimethylacetamide (DMAc). The Cyrene bicyclic, chiral, seven-membered heterocyclic cycloalkanone does not contain any chlorine, sulfur, or nitrogen heteroatoms, while its production is energy-neutral, sustainable, and based on renewable cellulose waste.^30^ Cyrene production also emits up to 80% less CO_2_ than NMP or DMF.^31^ The ReSolute* plant of the Circa Group AS, with the support of the EU Horizon 2020, reached 1000 tonnes of Cyrene production in 2023, with an 80 000 tonne capacity projected by 2030.^32^ Although there have been previous reports of utilizing Cyrene for phase-inversion PVDF and poly(ether sulfone) membranes,^33^ there are no reports of (fluoro)polymer electrospun membranes produced by incorporating Cyrene. Only one recent study using a polyether block amide dissolved at high temperature with Cyrene has been identified,^34^ where electrospinning was conducted at an unusually high flow rate, resulting in fibers with inhomogeneity and a lack of open porous structure. Elevated flow rates are purportedly unmanageable to achieve sustained jet stability over extended durations, making it challenging to produce a self-supporting membrane. Understanding the capability of Cyrene to solubilize and produce thermally stable (fluoro)polymer solutions opens the way to substituting hazardous solvents for reproducible, stable, and sustainable nanofiber processing. Cyrene has also been reported as a high-performance solvent for the sustainable processing of graphene derivatives.^35^

Therefore, the primary objective of this work was to develop more sustainable membranes using green solvents, such as Cyrene, via the electrospinning technique. Herein, we report the processability of poly(vinylidene fluoride)-co-hexafluoropropylene (PVDF-HFP), a close analogue of PVDF, into nanofibrous membranes, by employing solvent systems containing Cyrene. Subsequently, we assess the morphophysicochemical and membrane distillation properties of composite PVDF-graphene oxide and graphene nanoplatelet-doped electrospun membranes. To promote the progress of sustainable polymer processing, we have also compiled a list of commercially available, green-manufactured solvents that were evaluated during this study. These solvents may be of interest to researchers exploring green solvent–polymer processing alternatives.

## Experimental Section

### Materials

Poly(vinylidene fluoride)-co-hexafluoropropylene (PVDF-HFP) pellets (CAS 9011–17–0, *M*_w_ = 400 000 g·mol^–1^) were purchased from Sigma-Aldrich (USA). Dihydrolevoglucosenone (Cyrene) ≥98.5% (CAS 53716-82-8), triethyl phosphate (TEP) ≥99% (CAS 78-40-0), (−)-methyl l-lactate ≥98% (CAS 27 871-49-4), dimethyl sulfoxide (DMSO) ≥99.9% (CAS 67-68-5), dimethyl carbonate ≥99% (CAS 616-38-6), 2-methyltetrahydrofuran ≥99.5% (CAS 96-47-9), dimethyl isosorbide (DMI) ≥99% (CAS 5306-85-4), sodium chloride ≥99.5% (CAS 7647-14-5), and tetraethylammonium bromide ≥98% (CAS 71-91-0) were purchased from Sigma-Aldrich (USA). Ethyl l-lactate ≥98% (CAS 687-47-8), glycerol triacetate ≥99% (CAS 102-76-1), y-butyrolactone ≥99%, and acetone ≥99.5% (CAS 67-64-1) were purchased from Thermo Fisher Scientific (USA). Ethanol 99.8% (CAS 64-17-5) and acetic acid (CAS 64-19-7) were supplied by Honeywell International (USA). 2-Propanol ≥99.7% (CAS 67-63-0) was acquired from VWR Chemicals (USA). Graphene nanoplatelets (GNP, item GC-750) were purchased from XG Sciences (USA). Graphene oxide powder (GO, item C864/GOP18002/Pw) was purchased from Graphenea (USA). Nylon membrane supports (manufacturer part no. 0464A00023), 47 mm in diameter, with an average pore size of 0.45 μm, were purchased from Thermo Fisher Scientific (USA).

### Solution Preparation and Electrospinning

Initially, multiple solvent systems (Table 1) were explored to solubilize PVDF-HFP at 10% (w/v) by magnetic stirring at 70 °C until clear homogeneous solutions were obtained. If there was successful dissolution, the gelation time was determined by removing the polymer solution from the magnetic stirrer. Extensive parametric studies were also conducted with the various solvent systems containing Cyrene for the ability to achieve nanofibers via electrospinning (Table 1). Following the identification of the solvent system that consistently produced nanofibers, the electrospinning parameters including the voltage, distance, polymer concentration, and flow rate were optimized. The key adjustment was increasing the polymer concentration to 15% w/v. Higher polymer concentrations enhance the stability of the formed Taylor cone by increasing the viscosity and surface tension of the solution at the orifice of the spinneret when subjected to the electric field, thereby stabilizing jetting and improving the fiber output. This stepwise increase in the polymer concentration is a common strategy in polymer processing to optimize the fiber morphology, diameter, and overall production efficiency. To prepare the 0.5 and 1.0% (w/w) doped polymer blends, GO or GNP powders were directly added to a 15 wt % polymer solution (Cyrene:DMSO:DMC) and were vigorously magnetically stirred overnight and then ultrasonicated for an additional 15 min before use. The graphene derivatives were added directly to the stable polymeric solutions, as Cyrene is known to be a high-performance solvent for graphene derivative processing.^34^

**Table 1:** Evaluating the Solubility, Thermal Stability, and Electrospinnability of PVDF-HFP in Various Solvent Systems Containing Cyrene^a^^,^^b^

solvent system	ratio (*v*/*v*)	solute stability (gelation time)	electrospinnability
Cyrene	1	gelates almost instantly (reversible after reheating at 45 °C)	
Cyrene/acetone	7:3	gelates within 30 min	
Cyrene/ethanol	7:3	gelates within 30 min	
Cyrene/IPA	7:3	gelates within 30 min	
Cyrene/AcOH	7:3	gelates within 30 min	
Cyrene/TEP	7:3	gelates within 3 h	
	5:5	stable for several days	unsuccessful
	+0.25% (w/v) TEAB	stable for several days	unsuccessful
	+0.50% (w/v) TEAB	stable for several days	unsuccessful
	5:5 + 15 μL sulfonic acid	stable for several days	unsuccessful
Cyrene/TEP/ethanol	5:2.5:2.5	stable for several days	unsuccessful
Cyrene/MeOH-Lac	7:3	gelates within 30 min	
	5:5	gelates within 10 min	
Cyrene/GTA	7:3	gelates within 30 min	
Cyrene/ethyl-Lac	7:3	gelates within 30 min	
Cyrene/acetone/ethanol	5:2.5:2.5	gelates within 30 min	
Cyrene/DMSO	5:5	stable for several days	inconsistent jetting/wet deposition
	+0.25% (w/v) TEAB	stable for several days	inconsistent jetting/wet deposition
	+0.50% (w/v) TEAB	stable for several days	inconsistent jetting/wet deposition
	+hot air stream	stable for several days	inconsistent jetting
Cyrene/TEP/DMSO	5:2.5:2.5	stable for several days	unsuccessful
	+0.25% (w/v) TEAB	stable for several days	unsuccessful
	+0.50% (w/v) TEAB	stable for several days	unsuccessful
Cyrene/TEP/methyl-Lac	5:2.5:2.5	gelates within 1 h 30 min	
Cyrene/GTA/ethyl-Lac	5:2.5:2.5	gelates within 15 min	
Cyrene/TEP/methyl-Lac	5:2.5:2.5	gelates within 20 min	
Cyrene/TEP/GTA	5:2.5:2.5	stable for several days	inconsistent jetting/wet deposition
Cyrene/TEP/acetone	5:2.5:2.5	stable for several days	inconsistent jetting/wet deposition
Cyrene/DMSO/ethyl-Lac	5:2.5:2.5	stable for several hours	inconsistent jetting/wet deposition
Cyrene/TEP/DMSO/acetone	4:2:2:1	stable for several hours	inconsistent jetting/wet deposition
Cyrene/DMSO/GTA	5:2.5:2.5	stable for several days	inconsistent jetting/wet deposition
Cyrene/TEP/ethanol	5:2.5:2.5	gelates within 15 min	
Cyrene/DMSO/ethanol	5:2.5:2.5	gelates within 15 min	
Cyrene/2-MeTHF/DMC	4:4:2	stable for several days	inconsistent jetting/wet deposition
Cyrene/DMI	5:5	gelates within 30 min	n/a
Cyrene/dimethyl isosorbide/DMC	4:4:2	stable for several days	inconsistent jetting/wet deposition
Cyrene/GBL	5:5	stable for several days	inconsistent jetting/wet deposition
Cyrene/2-MeTHF	5:5	gelates within 30 min	n/a
Cyrene/DMC	5:5	stable for several days	successful/very low output
Cyrene/DMSO/acetone	4:4:2	stable for several days	successful
Cyrene/DMSO/DMC	4:4:2	stable for several days	successful

a The polymer (10% w/v) was dissolved at 70 °C for 4 h under magnetic stirring. Polymer solubilization was evaluated at room temperature.

b Abbreviations: 2-MeTHF, 2-methyltetrahydrofuran; AcOH, acetic acid; DMC, dimethyl carbonate; DMI, dimethyl isosorbide; DMSO, dimethyl sulfoxide; Ethyl-Lac, ethyl L-lactate; GBL, y-butyrolactone; GTA, glycerol triacetate; IPA, 2-propanol; MeOH-Lac, (−)-methyl L-lactate; NaCl, sodium chloride; TEAB, tetraethylammonium bromide; and TEP, triethyl phosphate.

Electrospinning experiments were conducted using a conventional apparatus consisting of a microfluidic infusion syringe pump (Aladdin-220, World Precision Instruments, UK), 0–25 kV positive and 0–10 negative potential power supplies (PWRSUP-BSE, Excellent Innovate Electronics, China), an aluminum plate (thickness 3 mm, 200 (h) × 200 (w) mm), and blunt 21 Gauge (inner diameter 0.514 mm) needles. The setup was placed within a chamber where the temperature was monitored and the humidity was controlled using a dehumidifier. The fibers were collected directly onto nylon (0.45 μm) supports mounted on the collector’s surface. Electrospinning was conducted from a 15 cm working distance, at a 15 μL min^–1^ flow rate, by applying a potential difference of 21.5 kV (+16/–5.5 kV) between the spinneret and the collector. Each experiment was conducted for 5 h. All experiments were performed at a chamber relative humidity of 30–45% and a temperature of 19–22 °C. The produced membranes were placed in an oven at 40 °C for 24 h.

### Membrane Liquid Entry Pressure and Zeta Potential

Liquid entry pressure (LEP) measurements were carried out using a GVS Filter Technology setup. The membrane cell was a stainless-steel filter holder connected to a reservoir filled with distilled water. Compressed air was used to gradually pressurize the water using a precision pressure regulator. The LEP value was taken as the applied pressure at which the first water droplet was observed to permeate through the membrane.

Zeta potential analyses were carried out with a SurPASS3 (Anton Paar) electrokinetic analyzer. The zeta potential at the membrane interface with a 5 mmol L^–1^ KCl aqueous solution was measured in the pH range from 8 to 2 (acid scan) at room temperature through its streaming potential measurement.^36^

### Vacuum Membrane Distillation

For vacuum membrane distillation (VMD) tests, the membrane module consisted of a double-jacket reservoir in which the membrane was allocated between two chambers (feed and permeate side). The reservoir, equipped with a mechanical stirrer, was filled with the feed solution (distilled water or 0.6 M NaCl solution) that was put in contact with the membrane surface (34.2 cm^2^). A digital circulating bath (Thermo Electro Corporation, HAAKE P5, Thermo Fisher Scientific, Rodano, Italy) was used to maintain the feed solution at the desired temperature (40, 50, and 60 °C). The vacuum on the permeate side (about 2 mbar) was created by means of a vacuum pump (Edwards XDS 5, Cinquepascal) and controlled by a digital vacuum meter (A921, Cinquepascal). The vapor permeate was condensed and collected in a cold trap immersed in liquid nitrogen. In the case of NaCl solution, the feed and permeate conductivity was analyzed by using a Thermo Scientific Orion Star A215 conductivity meter. The schematic illustration of the VMD setup employed is depicted in Figure S1 of the Supporting Information.

The water vapor flux (*J*) through the membranes was calculated by the following equation
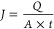
1where *Q* is the mass of the permeate (kg), *A* is the membrane area (m^2^), and *t* is the time (h).

Salt rejection (*R*) was calculated according to the following equation
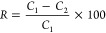
2where *C*_1_ is the feed conductivity (mS/cm), and *C*_2_ is the permeate conductivity (mS/cm). Further characterization details can be found in the Supporting information.

## Results and Discussion

### Green Solvent Screening

A series of green solvent systems were screened for their potential electrospinnabililty of PVDF (Table 1). Cyrene and triethyl phosphate, (−)-methyl l-lactate, ethyl l-lactate, glycerol triacetate, *y*-butyrolactone, dimethyl sulfoxide, acetone, ethanol, 2-propanol, acetic acid, dimethyl carbonate, 2-methyltetrahydrofuran, and dimethyl isosorbide were evaluated toward their ability to produce stable and homogeneous 10% w/v PVDF-HFP solutions at room temperature. As the electrospinning process necessitates a stable and homogeneous polymer solution at room temperature, achieving a stable Taylor cone and jetting requires optimization of various processing and ambient parameters.^37^ These requirements significantly increase the challenge of identifying suitable green solvents for electrospinning membranes compared to techniques such as phase inversion. Following the identification of solutions that formed stable, homogeneous mixtures at room temperature, these solutions were visually assessed for their ability to maintain a viscous flow without undergoing gelation, and their electrospinnability was evaluated and optimized (Table 1). Moreover, the green solvents identified in Table 1 have applications beyond the development of PVDF-HFP electrospinning membranes investigated in this study. Their potential extends to the evaluation of electrospinnability in a broader class of readily electrospinnable polymers that are currently tethered to or limited by the utilization of more environmentally and occupationally hazardous solvents during processing.

In the selection of solvent systems for the electrospinning of PVDF-HFP, Hansen solubility parameters (HSPs) were considered to ensure an optimal match between the polymer and the solvent. The HSP is based on three key components: dispersive (δ_d_), polar (δ_p_), and hydrogen (δ_h_) bonding. By comparing the HSP values of the polymer and the solvent, we can predict the solubility and compatibility, which is crucial for creating stable and homogeneous electrospinning solutions.

Cyrene was chosen as the primary solvent for this study due to its exceptional balance between solubility and sustainability. From a solubility perspective, Cyrene HSP values (δ_d_ = 18.0, δ_p_ = 13.5, δ_h_ = 7.3) are closely aligned with those of PVDF-HFP (δ_d_ = 18.2, δ_p_ = 12.3, δ_h_ = 7.1),^33^ indicating a good solubilization of the polymer in the used green solvent.

Applying the solubility distance equation
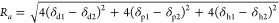
3we evaluated the Hansen solubility distance (*R*_a_) for each solvent system relative to PVDF-HFP.

The calculated solubility distance (*R*_a_ = 2.01) indicated a high degree of affinity between Cyrene and PVDF-HFP, ensuring effective polymer dissolution, which is critical for stable electrospinning. This strong compatibility promoted uniform nanofiber formation during the electrospinning process, making it an ideal solvent for achieving a consistent membrane morphology.

To enhance the electrospinnability and processing of PVDF-HFP, Cyrene was combined with DMSO and dimethyl carbonate (DMC) as cosolvents. Although these solvents show higher solubility distances (*R*_a_ = 5.16 for DMSO and 10.32 for DMC), their inclusion improved the electrospinning process by preventing gelation and ensuring a stable jetting behavior. The combination of Cyrene with DMSO and DMC thus optimized the solvent system’s performance, balancing solubility and process stability.

### Membrane Chemical and Physical Characterization

**Figure 1:**
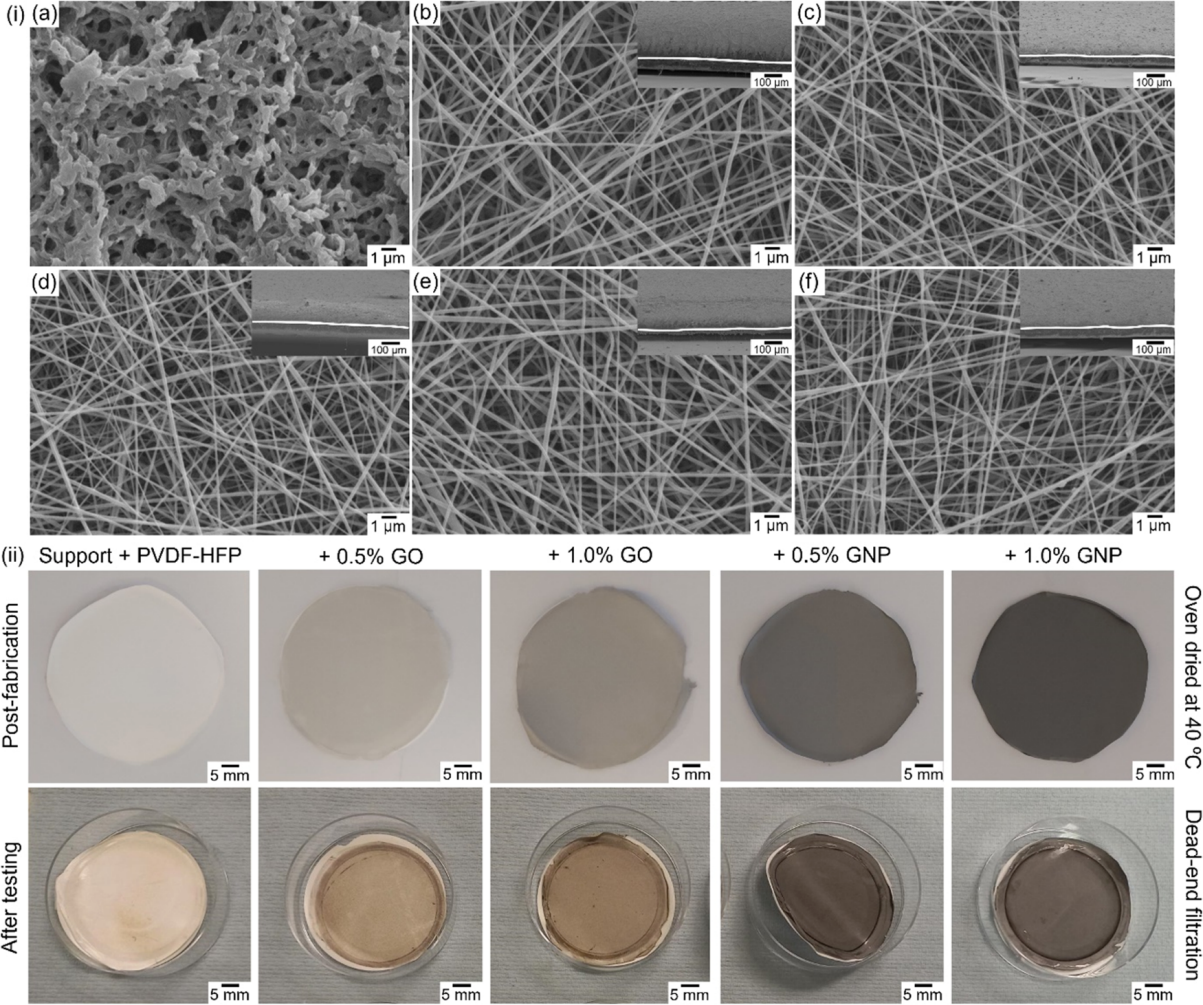
Morphological properties of the PVDF-HFP pristine and doped electrospun membranes produced on nylon supports. (i) SEM micrographs of (a) the nylon support, (b) pristine PVDF-HFP, (c) 0.5% GO, (d) 1.0% GO, (e) 0.5% GNP, and (f) 1.0% GNP NFs. The top right-hand side micrographs depict cross-sections of the deposited nanofibers and nylon support. (ii) Photographs of the pristine and doped membranes post-fabrication and after testing, depicting evident color differences proportional to the amount of GO and GNPs introduced into the polymer blends.

Following the selection of the most electrospinnable solvent system (Cyrene/DMSO/DMC, 4:4:2 v/v) and the electrospinning parameters being optimized, solutions were prepared for pristine PVDF-HFP and graphene-doped membranes (Figure 1). In combination with Cyrene, DMSO and dimethyl carbonate (also a green solvent) were used as cosolvents. DMSO is often defined as a greener alternative to traditional solvents^38^ or even a green solvent^39^ with no hazard classification. DMC has been employed as a volatile alternative solvent to traditional acetone, generally used in electrospinning solutions, thanks to its benign properties being readily biodegradable and nontoxic.^40^ All of the solvents used for the dope solution formulation were, therefore, characterized by a favorable eco-toxicological profile.

Graphene nanoplatelets (GNPs) and graphene oxide (GO) were incorporated to assess their impact on the electrospun membranes’ surface properties and ability to act as a distillation membrane for water desalination. Recognizing the interplay between the dopant behavior and the effect of the nanomaterial concentration within the membranes, blended solutions containing 0.5 and 1.0 wt % of both GO and GNPs were formulated and evaluated, using the green solvent system.

The polymer–solvent system was successfully employed to deposit a nanofibrous layer of ca. 100–150 μm thickness onto nylon support membranes (nominal pore size 0.45 μm), with and without the addition of the dopant nanomaterials. Visual confirmation of the incorporation of GO and GNPs was achieved through a gradual color change. Pristine membranes solely containing PVDF-HFP appeared white, whereas those containing the nanomaterials displayed a color shift toward different shades of gray. The intensity further increased with the dopant concentration (0.5–1.0 wt %), with darker membranes signifying higher loading (Figure 1(ii)). This initial observation suggests the successful integration of the nanomaterials into the fibrous structure.

Scanning electron microscopy (SEM) micrographs (Figure 1(i)) revealed a homogeneous surface composed of fine nanofibers with an average diameter between 75 and 100 nm (Table 2, Figures S2–S5). Figure 1(i) additionally provides an SEM micrograph of the cast nylon support membrane’s morphology and cross-sectional images of the integrated nanofibrous coating.

**Table 2:** Morphological and Functional Characterization of Electrospun Membranes: Average Fiber Diameter (*n* = 90), Average Pore Size, Porosity, Static Water and NaCl Solution Contact Angle (*n* = 3), and Polymer Solution Conductivity^a^

group	fiber diameter (nm)	pore size (μm)	porosity (%)	LEP (bar)	water contact angle (deg)	NaCl contact angle (deg)	solution conductivity (μS cm^–1^)
PVDF-HFP	94 ± 21	0.6 ± 0.1	68 ± 2	1.1 ± 0.3	134 ± 1	129 ± 5	5.7
PVDF-HFP + 0.5% GO	80 ± 21	0.6 ± 0.1	70 ± 5	1.4 ± 0.1	134 ± 4	134 ± 9	6.0
PVDF-HFP + 1.0% GO	79 ± 21	0.6 ± 0.1	89 ± 1	1.6 ± 0.3	139 ± 1	142 ± 3	7.2
PVDF-HFP + 0.5% GNP	89 ± 24	0.7 ± 0.2	86 ± 1	2.1 ± 0.1	135 ± 2	130 ± 3	50
PVDF-HFP + 1.0% GNP	74 ± 18	0.5 ± 0.1	78 ± 1	2.4 ± 0.2	136 ± 1	128 ± 3	80

a The data are presented as mean ± SD.

The fiber diameter distributions of pristine PVDF-HFP and its nanocomposite formulations with GO and GNP demonstrate notable differences in fiber diameters (Figure S5). The mean fiber diameter of pristine PVDF-HFP was 93.62 ± 21.42 nm, while the addition of 0.5 and 1.0% GO reduced the diameters to 80.74 ± 21.74 and 79.31 ± 21.04 nm, respectively. These reductions occurred despite only a minimal increase in the solution conductivity for the GO-containing specimens (from 5.7 to 6.0 and 7.2 μS cm^–1^). The finer fibers with a narrower distribution are likely a result of subtle molecular interactions between the GO sheets and the polymer chains, altering chain entanglement and jet stability during electrospinning.

In comparison, GNP composites exhibited a reduction in the fiber diameter affected by the concentration of the dopant within the solution, with mean diameters of 89.50 ± 24.15 nm for 0.5% GNP and 74.23 ± 18.40 nm for 1.0% GNP. These changes correlated with a significant increase in the solution conductivity (50 μS cm^–1^ for 0.5% GNP and 80 μS cm^–1^ for 1.0% GNP), likely enhancing electrostatic stretching forces during jet formation and producing finer fibers. Fiber distribution histograms and additional micrographs of the electrospun membranes can be found in the Supporting information (Figures S2–S5).

Despite these modifications, the overall morphology remained consistent, indicating that the nanofillers did not adversely impact the electrospinning process or the uniformity of the fibers. Notably, there were no visible signs of GO or GNP on the surfaces of individual fibers nor were any clusters evident within the membrane. The homogeneous distribution of the nanofillers suggests that the fiber morphology remained undisturbed, further indicating a uniform dispersion of the nanofillers throughout the fiber core rather than the formation of surface irregularities. The interplay of interfacial energy and differential charge distribution between the nanoparticles and the polymeric solution likely contributes to the consistent Taylor cone and jet formation during spinning,^37,41^ further suggesting that GO and GNPs are integrated within the core of the composite fiber structure.

The pristine PVDF-HFP membrane exhibited a porosity of 68% (Table 2). The introduction of GO and GNPs led to a porosity increase (up to 89%). This increase suggests the formation of a more open and less dense polymeric matrix, even though the average pore size remained relatively unchanged (Table 2). The observed increase in porosity can be directly linked to the electrospinning process and the effect of GO and GNPs on the nanofiber morphology. When GO and GNPs are added to the polymer solution, they influence the electrospinning parameters, resulting in finer nanofibers. Specifically, the presence of GO and GNPs increases the solution’s electrical conductivity,^43^ which, in turn, leads to an accelerated stretching of the polymer jet during electrospinning. This increased stretching reduces the average diameter of the nanofibers, enhancing the overall porosity. Moreover, GO and GNPs act as nanofillers that interact with the polymer matrix, disrupting the dense packing of polymer chains and creating an additional free volume.

The addition of nanofillers at these low concentrations did not significantly alter the water contact angle, leading to only a modest increase in hydrophobicity. This may be attributed to the surface properties of the membrane being primarily governed by the polymer matrix, with the filler material embedded within, rather than exposed on the nanofibers’ surface. Similar observations were also observed when measuring the contact angle with NaCl solution (Table 2).

**Figure 2:**
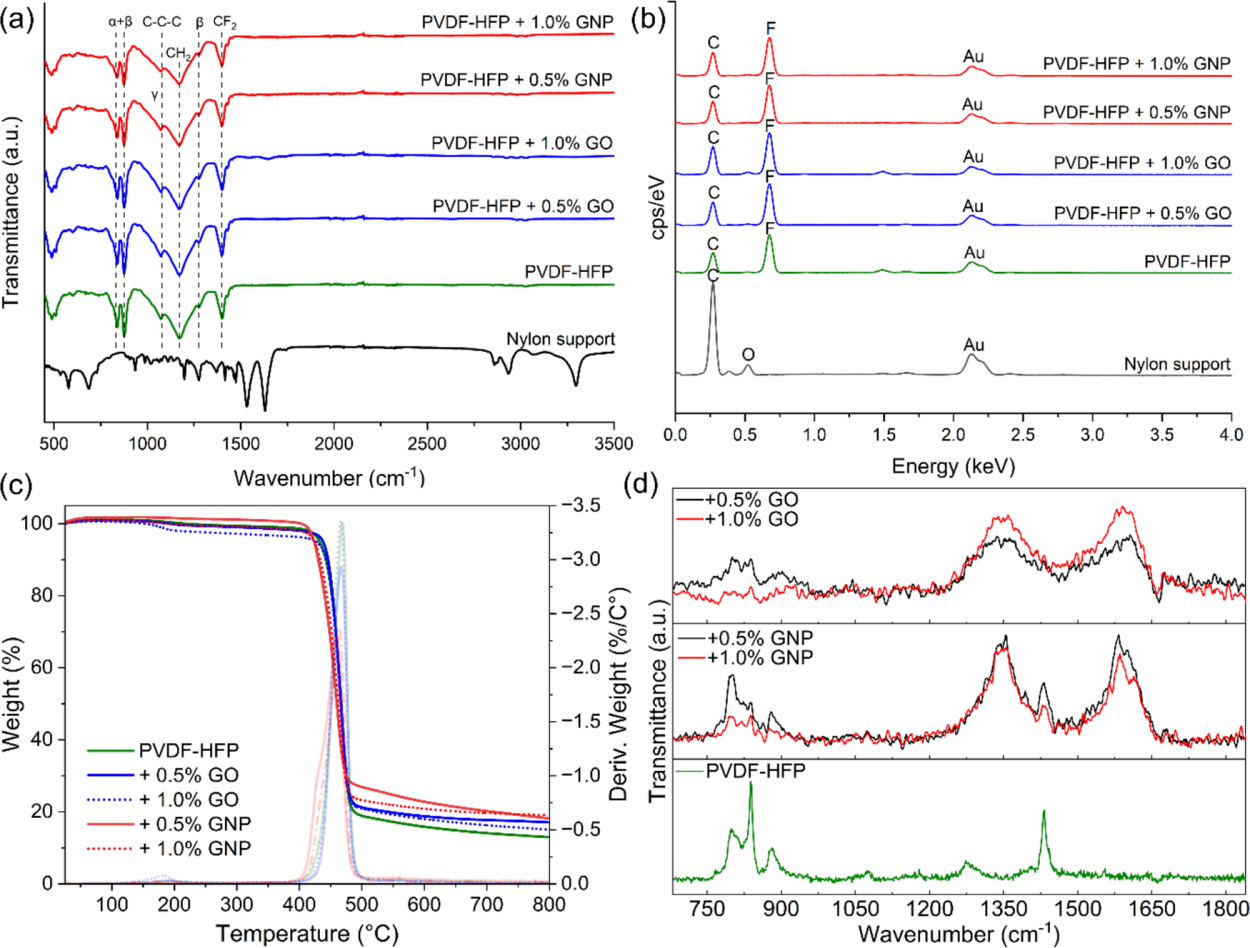
Chemical and thermal properties of the pristine and GO- and GNP-doped electrospun membranes. (a) ATR-FTIR spectra; (b) representative EDX elemental mapping graphs; (c) TGA and DTG contours of the nanofibrous membranes under an argon atmosphere; and (d) Raman spectra.

PVDF-HFP exhibits several crystalline phases (α, β, and γ). Processing parameters and solvent selection during electrospinning have been shown to influence the degree of crystallinity and the formation of specific phases. PVDF-HFP is a copolymer composed of vinylidene fluoride (VDF) and hexafluoropropylene (HFP) monomers. FTIR analysis (1 cm^–1^ resolution, 126 scans) revealed no significant differences between pristine and graphene-doped membranes (Figures 2a, S6, S7, and Table S1). This further suggests the entrapment of nanofillers within the cores of the fibers during electrospinning.

The α phase of PVDF-HFP was identified by a characteristic peak at 837 cm^–1^, assigned to the α-(CH_2_CF_2_)*_n_* skeletal vibration of the VDF moiety.^42^ The peak at 1175 cm^–1^ corresponds to the α-CF_2_ symmetric stretching mode of the PVDF segment.^43^ The β phase was identified by the sharp peak at 880 cm^–1^, attributed to the β-(CH_2_CF_2_)*_n_* skeletal vibration of the PVDF unit.^44^ A signature peak for the β phase is the CF*_2_* symmetric stretching mode of the HFP moiety, appearing around 1274 cm^–1^.^45^ Additionally, the peaks at 1274, 1175, and 1073 cm^–1^ are associated with the bending and wagging vibrations of CF bonds in the HFP and VDF units.^46^ The weak peak at 1073 cm^–1^ further suggests the presence of a γ phase, generally assigned to both PVDF and HFP units.^47^

EDX elemental analysis (information depth of about 10 nm) (Figure 2b) of the electrospun membrane surfaces revealed the expected dominant presence of fluorine (F) and carbon (C), corresponding to the polymer backbone. The nylon support membrane, as expected for a polyamide, showed populations of oxygen (O) and C. There were no consistent differences in the fluorine-to-carbon (F/C) ratio among the pristine and nanomaterial-doped membranes.

Thermogravimetric analysis (TGA) under argon was conducted to elucidate the thermal behavior and relative stability of the PVDF-HFP nanofibrous membranes and to explore the influence of GO and GNP incorporation (Figure 2c). All compositions exhibited a marginal initial weight loss (all <5%) around 100 °C, likely due to the evaporation of residual moisture. A gradual weight loss was then observed until the onset decomposition temperature of 420 °C, followed by a DTG peak at 465 °C corresponding to approximately 80% weight loss, signifying the decomposition of the PVDF-HFP polymer. The presence of dopants (GO and GNPs) appeared to have a modest effect on the thermal stability. Composites containing GNPs and GO exhibited a slower weight loss rate and a slightly lower total weight loss (77–78% for GNPs and 71–74% for GO) compared to pristine membranes. This observed decrease in weight loss for GNP-containing membranes could be attributed to its good thermal conductivity, potentially allowing for more uniform heat distribution. For GO-containing membranes, the oxygen-containing functional groups might create a physical barrier or interact with the PVDF-HFP polymer chains, hindering their decomposition to some extent. These results align with previously published works.^46,48^

Raman spectra of the PVDF-HFP membranes are depicted in Figure 2d, which reveal peaks at 838 and 798 cm^–1^, assigned to the β/γ and γ phases, respectively.^49^ The peak at 881 cm^–1^ suggests the coexistence of α, β, and γ phases.^50^ The sharp peak at 1432 cm^–1^ can be attributed to CH_2_ bending vibrations of the PVDF phases.^50^ Notably, the absence of significant peaks between 1350 cm^–1^ (D band) and 1580 cm^–1^ (G band) is indicative of the absence of detectable sp^2^-bonded carbon features, thus making it a good determinant to evaluate the presence of GO or GNPs in the composite membranes. The composite membranes containing GO and GNPs displayed additional features, confirming the successful incorporation of graphene-based nanomaterials into the nanofiber structure. Notably, the presence of a D band at around 1336 cm^–1^, characteristic of sp^2^ carbon structures found in GO, is observed.^51^ Its broader and less defined presence could be attributed to the disruption of the perfect hexagonal lattice structure of graphene by oxygen-containing functional groups (epoxides, hydroxyls, and carbonyls) within GO.^51,52^ Conversely, the G band at ∼1578 cm^–1^ indicates the presence of in-plane ordered sp^2^ domains within incorporated GO.

The GNP-containing membranes exhibited a more intense and well-defined D band at 1355 cm^–1^ compared to GO. This aligns with GNPs possessing larger and more ordered sp^2^-bonded carbon domains due to their reduced defect density.^52^ Similarly, a higher G band intensity at ∼1582 cm^–1^ reflects a higher degree of in-plane order and a greater fraction of sp^2^ carbons within GNPs compared to GO. These results confirm the presence of the nanomaterials in the composite structures, confirming the visual observations made during development.

The liquid entry pressure (LEP) is defined as the minimum entry pressure before water penetrates the membrane pores, which depends on the membrane maximum pore size and the membrane’s hydrophobicity as defined by the Young–Laplace equation.^53^ The hybrid membranes’ LEP values (Table 2) slightly increased as the content of GO and GNPs into the membrane increased. Since the pore size of the membranes was relatively constant (Table 2), the improvement of LEP values can be mainly ascribed to the improvement of the membrane surface hydrophobicity, as evidenced by contact angle results. The improvement of the LEP without a reduction of the membrane pore size can be beneficial in enhancing the water flux of the hybrid membranes in MD processes.^23^ The mean pore size of the membranes was in the microfiltration range (0.5–0.7 μm) without being greatly influenced by the addition of nanofillers (Table 2).

**Figure 3:**
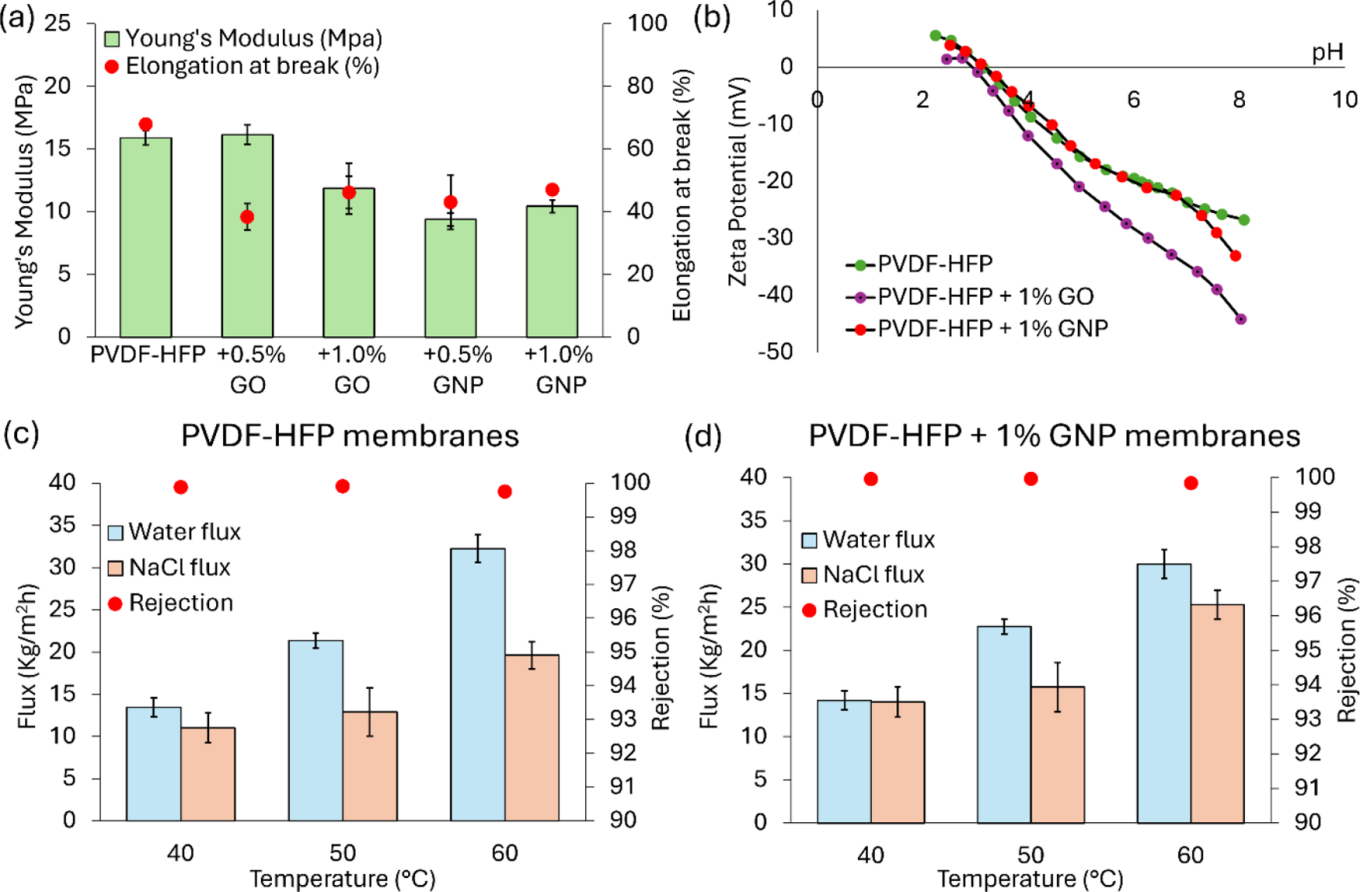
Membrane characterization, summarizing the key structural, mechanical, and performance properties of the fabricated membranes. (a) Mechanical properties derived from the stress–strain tensile strength evaluation presenting Young’s Modulus and elongation at break. (b) Zeta potential measurements of developed membranes as a function of pH. (c) Trend of the permeate fluxes and salt rejection as a function of the feed temperature during the VMD tests for the pristine PVDF-HFP membrane. (d) Trend of the permeate fluxes and salt rejection as a function of the feed temperature during VMD tests for the PVDF-HFP + 1% GNP membrane.

The addition of both nanofillers caused a decrease in the membranes’ mechanical properties, making them slightly more fragile with respect to the PVDF-HFP pristine membrane (Figure 3a). In this regard, the literature reports contradictory results since graphene-based nanomaterials, on the basis also of their concentration, were reported to lead to both an improvement^54^ or a decrease^55^ of the membranes’ mechanical properties. In the present case, the Young’s modulus values decreased as the porosity increased (Figure 3a). A higher porosity, in fact, contains the numerous voids in the membrane, which represent weak points in its structure.^56^

The membrane surface charge density can change by the incorporation of nanofillers because of new and different functional groups exposed at the surface. Zeta potential measurements were performed on the membranes loaded with the highest concentration of nanofillers (1 wt %). The pristine PVDF-HFP membrane showed the characteristic trend of uncharged polymeric materials^57^ (Figure 3b). The membrane surface appears negatively charged due to the adsorption of hydroxide ions (originating from the self-ionization of water), reaching its isoelectric point (IEP) at a pH of 3.14^58^ with a zeta potential of −23.7 mV at a pH of 7. The same trend was observed for the PVDF-HFP + 1% GNP membrane that reached its IEP at the same pH of the pristine membrane since GNPs do not contain charged functional groups. The zeta potential of −23.7 at a pH of 7 can be, therefore, explained by the adsorbed hydroxide ions.

However, upon the addition of GO fillers, the PVDF-HFP + 1% GO membrane surface resulted in a more negatively charged surface due to the deprotonation, especially at higher pH values, of carboxylic (−COOH) and hydroxyl (−OH) groups (borne by GO) into −COO^–^ and −O^–^ negative groups.^59^ The PVDF-HFP + 1% GO membrane reached its IEP at pH 3 with a zeta potential of −35 mV at pH 7.

### Vacuum Membrane Distillation

Pure water vacuum membrane distillation (VMD) tests were conducted to study the influence of the feed temperature on the permeation flux without the phenomena of the concentration gradient and mass transport resistance in the liquid phase present in the case of salty solutions. VMD tests were carried out on the pristine PVDF-HFP and PVDF-HFP + 1% GNP membranes. Despite their apparent hydrophobic nature, the membranes prepared with GO could not be successfully tested in VMD because of the wetting phenomena. This can be related to the lower LEP values measured for the PVDF-HFP + GO membranes (Table 2) as a consequence of the presence, on their surface, of polar −COOH and −OH groups characteristic of GO nanoparticles. The water vapor flux for the PVDF-HFP pristine membrane increased from 13.4 to 32.2 kg/m^2^ h as the feed temperature increased from 40 to 60 °C (Figure 3c). As the feed temperature increased, a higher vapor pressure was created on the feed side, resulting in a higher partial pressure gradient between the feed and permeate sides (increased driving force).^60^ For the tests carried out with the salty solution, a decrease of the permeate flux (up to almost 40%) was observed as a consequence of the lower water activity.^61^ The salt rejection was always higher than 99.7% at all investigated temperatures, reaching its maximum at 50 °C (99.9%).

The same temperature dependency for both flux and rejection was found for the PVDF-HFP + 1% GNP membrane (Figure 3d). For this membrane, despite the lower pore size, the measured water vapor flux was between 14 and 30 kg/m^2^ h, which was comparable to that of the pristine PVDF-HFP membrane. The permeate flux measured with the NaCl solution was notably higher (with an increase of 18–22%) than that of the PVDF-HFP pristine membrane at all investigated temperatures. This improvement in the flux for the salt solution is due the addition of GNPs into the membrane, which led to an increase of its hydrophobicity and LEP.^21,22^

In this study, it was observed that the inclusion of GNPs not only slightly enhanced the membrane’s hydrophobicity and LEP but also significantly increased its porosity, from 68 to 78%. This increase in porosity directly contributes to the improvement in the flux by reducing the mass transport resistance as a higher porosity allows for greater vapor transport through the membrane. The improvement in porosity also enhances vapor diffusion without compromising the membrane’s rejection performance. While hydrophobicity itself does not directly increase the flux, it plays a critical role in preventing pore wetting, thus maintaining the membrane’s ability to reject liquid water and allowing it to operate at higher driving forces without compromising the performance. The higher LEP reduces the risk of pore wetting, which would otherwise hinder vapor transport and lower the flux.

Moreover, the drop in the permeate flux between salty solution and pure water was much smaller for the PVDF-HFP + 1% GNP membrane compared to the pristine PVDF-HFP membrane, whose flux decreased by up to 40% when NaCl solution was fed to the membrane. The lower susceptibility of the PVDF-HFP + 1% GNP membrane to NaCl can also be linked to the presence of GNPs, which limited wetting.^62^ The PVDF-HFP + 1% GNP membrane displayed a higher salt rejection (up to 99.97%) at all investigated temperatures with respect to the pristine membrane. This improvement can be related to an increase of the tortuous pathway as a consequence of GNP incorporation.^63,64^ The presence of graphene-based materials within the membrane, in fact, disrupts the linearity of the pore pathways, introducing a more complex and winding route for vapor diffusion.^65^ The increased tortuosity typically corresponds to an increase in the membrane’s LEP which helps prevent liquid water (and the dissolved salts) from passing through the membrane under higher driving forces, allowing only vapor to pass through.^66^

Furthermore, PVDF-HFP + 1% GNP membranes exhibited a much higher stability during the MD tests for at least 28 h (consecutive tests carried out on the same membrane sample), while the PVDF-HFP pristine membranes were much more susceptible to flooding, which occurred in comparison after only ca. 6 h (when testing with the salty solution). Such improvement in stability, in terms of the flux, rejection, and long-term resistance in MD, has been also observed in literature for other polymeric membranes loaded with GNPs produced utilizing DMF, etc. (Table 3).^64^ The trend of permeate fluxes as a function of the feed temperature during VMD tests for the pristine PVDF-HFP and PVDF- HFP + 1% GNP membranes is displayed in Figure S8 of the Supporting Information.

**Table 3:** Comparative Overview of Graphene-Based PVDF Membranes Utilized for the Desalination of Water via Membrane Distillation^a^

polymer:filler	conc. (wt %)	spinning solvent	WCA (deg)	*D* (nm)	flux (kg/ m^2^ h)	*T*_in_ (°C)	LEP (kPa)	*R*_NaCl_ (%)	ref
PVDF-HFP:-	15:0	cyrene/DMSO/DMC	134	94	∼19.60	60	110	99.75	this work
PVDF-HFP:GNP	15:1	cyrene/DMSO/DMC	136	74	∼25.29	60	240	99.84	this work
PES/PVDF:GNP	17.25/5.75:2	DMF/NMP	132	356	∼19.35	65	85		(21)
PVDF:GNP	14:2	DMF/NMP	141	352	∼19.77	65	95		(21)
PVDF-HFP:GNP	18:5	DMF/acetone	162	378	∼22.90	60	186	100	(22)
PVDF-HFP:rGO	15:0.15	DMAc/acetone	139	166	∼20.37	50–70	103	99.97	(25)
PVDF-HFP:rGO	15:0.15	DMAc/acetone	139	171	∼27.94	35–65	105	97–99	(24)
PVDF-HFP:rGO-POTS	15:0.15	DMAc/acetone	155	184	∼27.94	35–65	120	99.99	(24)
PVDF:FTES-GO	14:4	DMF/acetone	141	51	∼36.40	50	234	99.90	(23)

a Abbreviations: WCA (water contact angle), *D* (average fiber diameter), *T*_in_ (inlet temperature), LEP (liquid entry pressure), *R*_NaCl_ (rejection of sodium chloride at 3.5 wt %), and NR (not reported).

In this study, Cyrene contributed to maintaining the membrane efficiency, primarily due to its excellent solvent properties for dissolving PVDF and its ability to produce uniform, defect-free nanofibers during the electrospinning process. Additionally, the physical and chemical properties of Cyrene helped to maintain the pore structure and mechanical strength of the membranes, which are critical for the performance in membrane distillation. When comparing membranes produced in this present study with comparable membranes fabricated with DMF (Table 3) with the assumption of comparable solvent use (as the polymer concentrations are similar), ca. 4.22 kg of CO_2_ would be emitted per kg of DMF used in comparison to 0.844 kg of CO_2_ emitted per kg of Cyrene.^32,64^ Therefore, our results confirm that sustainable production of PVDF membranes using green solvents like Cyrene is feasible without sacrificing the membrane performance, paving the way for more ecofriendly industrial practices.

### Technoeconomic Assessment

To evaluate the economic viability of incorporating green solvents in membrane production via electrospinning, a comparative technoeconomic analysis was conducted using Monte Carlo simulations to address inherent uncertainties in the model.^67^ The key economic indicator used was the total production cost (TPC) for fabricating 1 m^2^ of membrane with a 100 μm thickness and 60% porosity, as detailed in this study. The TPC encompasses the costs of raw materials, utilities, and fixed overheads. The fixed costs include labor, supervision, salary overhead, maintenance, plant overhead, royalties, and taxes. Table 4 presents a detailed breakdown of the raw material costs, while utilities and fixed costs were estimated using established engineering methods commonly referenced in the literature.^67^ The analysis assumes the use of an industrial electrospinning machine, processing 0.09 kg/h of solution, which equates to an annual production capacity of 301,186 m^2^ of membrane.^68^

**Table 4:** Price of Raw Materials Used to Assess the Economics of Green Solvents

direct production costs (raw materials)	ref
a. PVDF = £45/kg	(69)
b. Cyrene = £190/kg	(29), (70)
c. DMSO = £38/kg	(71)
d. DMC = £8/kg	(72)
e. DMAc = £7.5/kg	(72)

**Figure 4:**
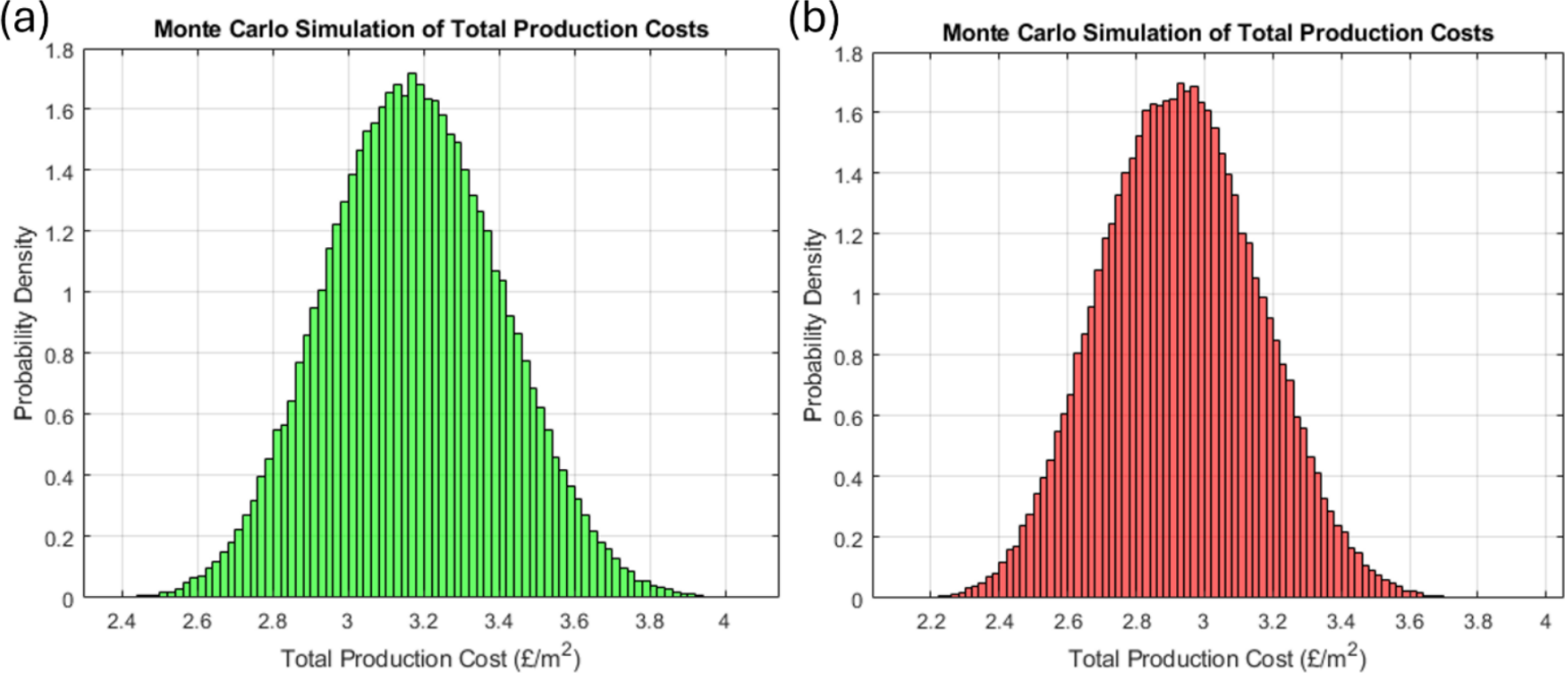
Probability distribution of the TPC for the production of electrospun membranes using (a) Cyrene/DMSO/DMC as the green solvent and (b) DMAc as the state-of-the-art.

The technoeconomic analysis aimed to compare the economic performance of PVDF-HFP membranes electrospun using two different solvent systems: a green solvent mixture of 4:4:2 Cyrene/DMSO/DMC at 15 wt % and the conventional DMAc solvent at the same polymer concentration. The probability distribution of the total production cost (TPC) for both systems reveals a modest increase in the mean TPC from £2.85 to £3.20 per m^2^, representing approximately a 10% rise in production costs when utilizing green solvents (Figure 4). It is important to note that the cost range aligns with commercial membranes and those found in the literature.^73,74^

The significant overlap in the probability distributions between the green solvent system and the DMAc-based system indicates that the risk associated with adopting green solvents is minimal concerning the TPC. This overlap suggests that while the mean cost rises slightly, the variation in cost outcomes remains comparable between the two systems, implying that the adoption of greener alternatives does not substantially increase economic risk from a production cost perspective.

Further sensitivity analysis shows that the price of the green solvent mixture would need to be eight times higher than that of DMAc for the mean TPC of the green system to match the 90th percentile of the conventional solvent’s cost distribution. This finding demonstrates that the economic feasibility of green solvents remains robust unless there is a dramatic surge in solvent prices, which is unlikely, given the current market trends toward increased availability and regulatory incentives for sustainable chemicals. Therefore, the green solvent system presents a viable alternative with minimal economic trade-offs, supporting its use in more sustainable membrane production processes.

## Conclusions

In this study, green electrospun PVDF-HFP membranes have been successfully produced for the first time using a Cyrene-containing green solvent system. GNP and GO fillers were also incorporated, at different concentrations, into the nanofibrous network to assess their impact on the membrane properties and performance when applied in membrane distillation for water desalination. Raman spectroscopy confirmed the effective integration of the graphene-based nanomaterials into the nanofiber structure, with uniform and defect-free nanofibers with an average diameter of 75–100 nm, obtained. All of the produced membranes exhibited a hydrophobic nature and a pore size in the microfiltration range (0.5–0.7 μm). The addition of graphene-based nanomaterials led to an improvement of LEP values with respect to the pristine PVDF-HFP membrane, which was much more evident for the membranes loaded with GNP. Vacuum membrane distillation tests, carried out at different temperatures, showed that the produced PVDF-HFP membranes were able to operate with a satisfying flux and high NaCl rejection with results comparable (or even better) to analogous PVDF electrospun membranes produced with traditional toxic solvents. The addition of GNP led to an improvement of the permeate flux, salt rejection, and membrane long-term stability. The development of graphene-based polymeric nanofibrous membranes using green approaches represents a significant advancement in the manufacturing of more sustainable membranes by the electrospinning technique. The combination of good performance characteristics and environmentally friendly solvents, alongside economic viability, aligns these membranes with environmental sustainability goals, minimizing the ecological footprint of the membrane manufacturing process.

## Supporting Information Available

Additional experimental details including the membrane distillation setup; SEM micrograph analysis; FTIR analysis; and further methodological details (PDF)
