# Supplementary material for: Cyrene-Enabled Green Electrospinning of Nanofibrous Graphene-Based Membranes for Water Desalination via Membrane Distillation
Source: ACS Sustain Chem Eng. 2024 Nov 25;12(49):17713–25. doi: 10.1021/acssuschemeng.4c06363 (PMC11632766; doi:10.1021/acssuschemeng.4c06363)
Supplement: Supplementary file 1 — sc4c06363_si_001.pdf [file sc4c06363_si_001.pdf]

Supporting Information

Cyrene-enabled Green Electrospinning of Nanofibrous Graphene-Based Membranes for Water Desalination Via Membrane Distillation

‡Antonios Keirouz[a], ‡Francesco Galiano[b], Francesca Russo[b], Enrica Fontananova[b], Bernardo Castro-Dominguez[a], Alberto Figoli[b], Davide Mattia[a], Hannah S. Leese[a]*

[a] Department of Chemical Engineering, Faculty of Engineering & Design, University of Bath, Bath, BA2 7AY, UK

[b] Institute on Membrane Technology, National Research Council of Italy (CNR-ITM), Via Pietro Bucci 17/C, 87036, Arcavacata di Rende, CS, Italy
‡contributed equally

*corresponding author: h.s.leese@bath.ac.uk

Table of Contents

Further Experimental details
Figure S1. Schematic illustration of the VMD set-up employed
Figure S2. SEM Micrographs of pristine PVDF-HFP electrospun membranes.
Figure S3. SEM micrographs of graphene oxide (GO)-doped PVDF-HFP electrospun membranes.
Figure S4. SEM micrographs of graphene nanoplatelets (GNP)-doped PVDF-HFP electrospun membranes.
Figure S5. Fiber diameter distribution histograms of electrospun membranes. Histograms illustrating the fiber diameter distributions of pristine PVDF-HFP and doped with graphene oxide (GO) and graphene nanoplatelets (GNPs).
Figure S6. ATR-FTIR spectra of a) the graphene oxide (GO) powder and composite PVDF-HFP + GO electrospun membranes and of b) the graphene nanoplatelets (GNP) powder and composite PVDF-HFP + GNP electrospun membranes.
Figure S7. FTIR comparative analysis of absorbance spectra of the composite PVDF-HFP + GNP or GO vs the corresponding nano-powders.
Table S1. Comparative analysis of FTIR peak positions and functional group assignments for graphene nanoplatelets (GNP) and electrospun PVDF-HFP-GNP composite membranes.
Table S2. Comparative analysis of FTIR peak positions and functional group assignments for graphene oxide (GO) and electrospun PVDF-HFP-GNP composite membranes.
Figure S8. Trend of the permeate water fluxes and salt fluxes as a function of the feed temperature during VMD tests for the pristine PVDF-HFP membrane (a,b) and PVDF- HFP + 1% GNP membrane (c,d)

Further Experimental details

Morphological assessment

A scanning electron microscopy (SEM) instrument, SU3900 (Hitachi-High Technologies, Japan) was used to appraise the electrospun membranes fiber morphology. The specimens were sputter-coated with a 10 nm gold layer to form a conductive surface using a Q150TS Plus sputter coater (Quorum, USA). High-resolution micrographs were attained at 15 kV accelerating voltage from a 10 mm working distance. Cross-sections were further captured to evaluate the interface between the nylon support and deposited fibers, by tilting the sample holder, enabling electron tomography. The mean fiber diameter was computed using the ImageJ software (v.1.8, National Institutes of Health, USA). A total of 90–120 fiber width values were measured from three micrographs per specimen.

Chemical and thermal characterisation

Energy dispersive X-Ray (EDX) elemental composition analysis was performed using a 170 mm^2 sensor size Ultim® Max EDS detector (Oxford Instruments, UK) attached to the SEM instrument. Elemental maps were collected using the AZtec software package.

The chemical composition of the surfaces was carried out via elemental mapping by energy-dispersive X-ray (EDX) spectroscopy using a 170 mm^2 Ultim Max EDX detector (Oxford Instruments, UK) attached to the SEM. The data were interpreted using the AZtec software package.

Fourier-transform infrared spectroscopy (FTIR) spectra were obtained using the iD7 ATR accessory of a Nicolet™ iS™ 5 infrared spectrometer (Thermo Fisher Scientific, USA). Each spectrum was collected in the wavenumber range of 4500–600 cm^{-1} and represents the average of 126 scan rates at a spectral resolution of 1 cm^{-1}. Raman spectra were collected using an inVia™ confocal Raman microscope (Renishaw, UK). The specimens were irradiated using a green line laser (wavelength 532 nm) set at 1or 5% (69 mW) power, where 10 s exposure with three accumulations point spectra were obtained under ambient conditions.

The water contact angle (WCA) and the NaCl contact angle of each electrospun membranes was determined using the OCA 25 optical contact angle measuring instrument using the SCA 20 software (Data Physics, UK). On each vacuumed dried membrane, a 5 µL droplet of deionised water was dispensed onto the surface, allowing it to settle for 30 s. The values were computed from snapshots using the contact angle add-on of the ImageJ software (v.1.8, National Institutes of Health, USA).

Thermogravimetric analysis (TGA) was performed using a SETSYS Evolution TGA (Armgate, Latvia). Specimens between 8-12 mg were loaded onto ceramic crucibles and heated from 30 °C to 800 °C at a 10 $K \cdot min^{-1}$ rate under constant argon flow. The data were computed using the Calisto Thermal Analysis

software to produce the weight-loss and differential weight-loss curves to evaluate thermal stability and the onset of thermal degradation (T_{deg}).

Pore size, mechanical properties, porosity

The mean flow pore diameter was evaluated by means of a capillary flow porometer (POROLUX^{TM} 1000, Porometer, IB-FT GmbH, 12277 Berlin, Germany). Each sample was soaked into Porewick (16 dyne/cm) as a liquid with a low surface tension. The pore size measurements were carried out on the electrospun membrane detached from the Nylon support.

Membranes mechanical properties were measured using a Zwick/Roell testing machine, single-column model Z2.5, equipped with a 50 N maximum load cell (BTCFR2.5TN-D09, Zwick/Roell). Porosity measurements were carried out by registering the weight of the membrane samples before and after their immersion for 24 h into kerosene used as a wetting liquid.

Membranes porosity was calculated according to the following equation:

$$\text{Porosity (\%)} = \frac{\frac{wt_w - wt_d}{\rho_k}}{\frac{wt_w - wt_d}{\rho_k} + \frac{wt_d}{\rho_p}} \times 100 \qquad \text{(Eq S1)}$$

where w_{tw} is weight of the wet membrane, w_{td} is the weight of the dry membrane, ρ_k is the kerosene density (0.786 g/cm^3) and ρ_p is the polymer density (PVDF: 1.77 g/cm^3).

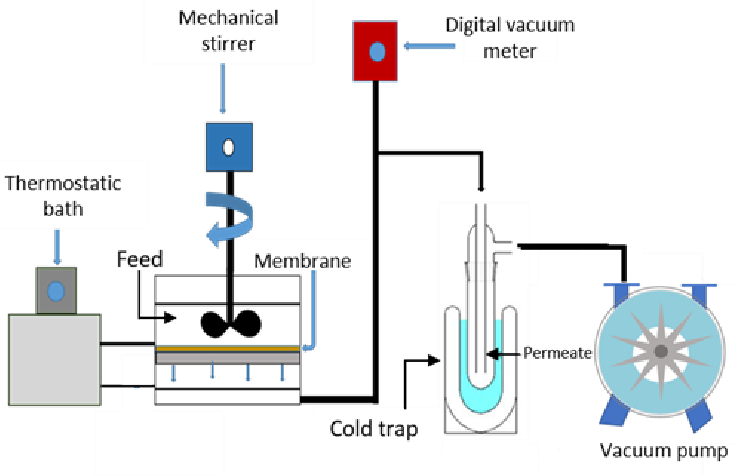

Figure S1. Schematic illustration of the vacuum membrane distillation set-up

Pristine PVDF-HFP

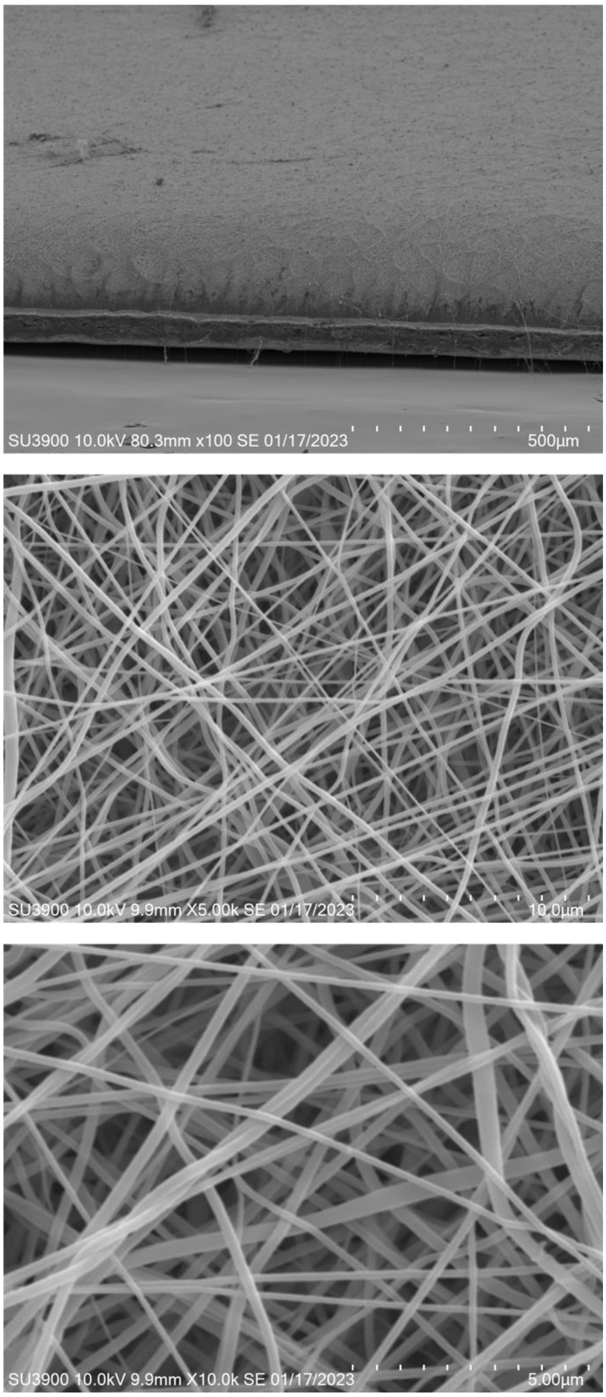

Figure S2. SEM Micrographs of pristine PVDF-HFP electrospun membranes. Uncropped, unmodified SEM micrographs illustrating the morphology of pristine PVDF-HFP fibers deposited onto a nylon-6 support substrate.

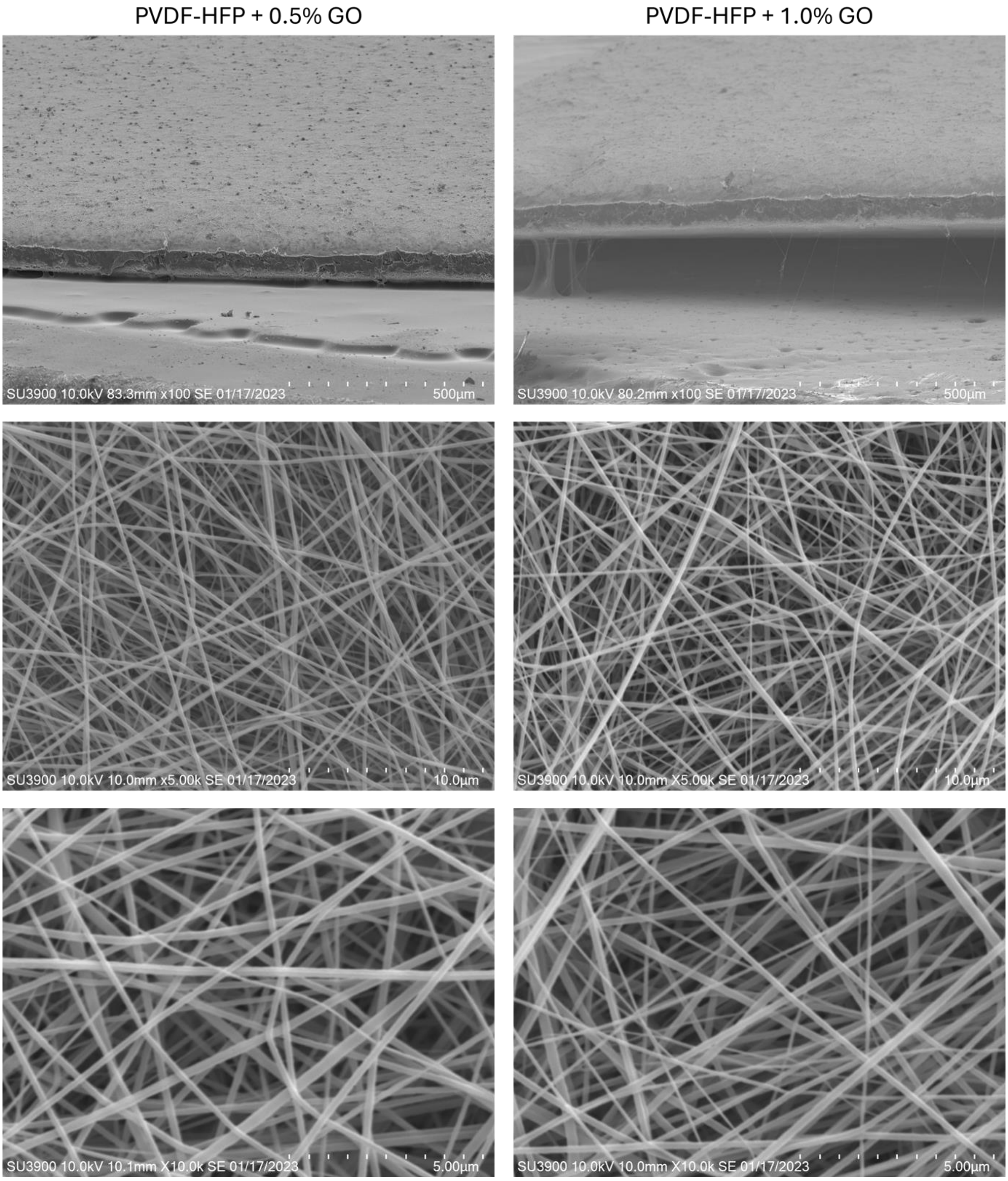

Figure S3. SEM micrographs of graphene oxide (GO)-doped PVDF-HFP electrospun membranes. Uncropped, unmodified SEM micrographs illustrating the morphology of PVDF-HFP fibers doped with 0.5 wt% and 1.0 wt% GO, deposited onto a nylon-6 support substrate.

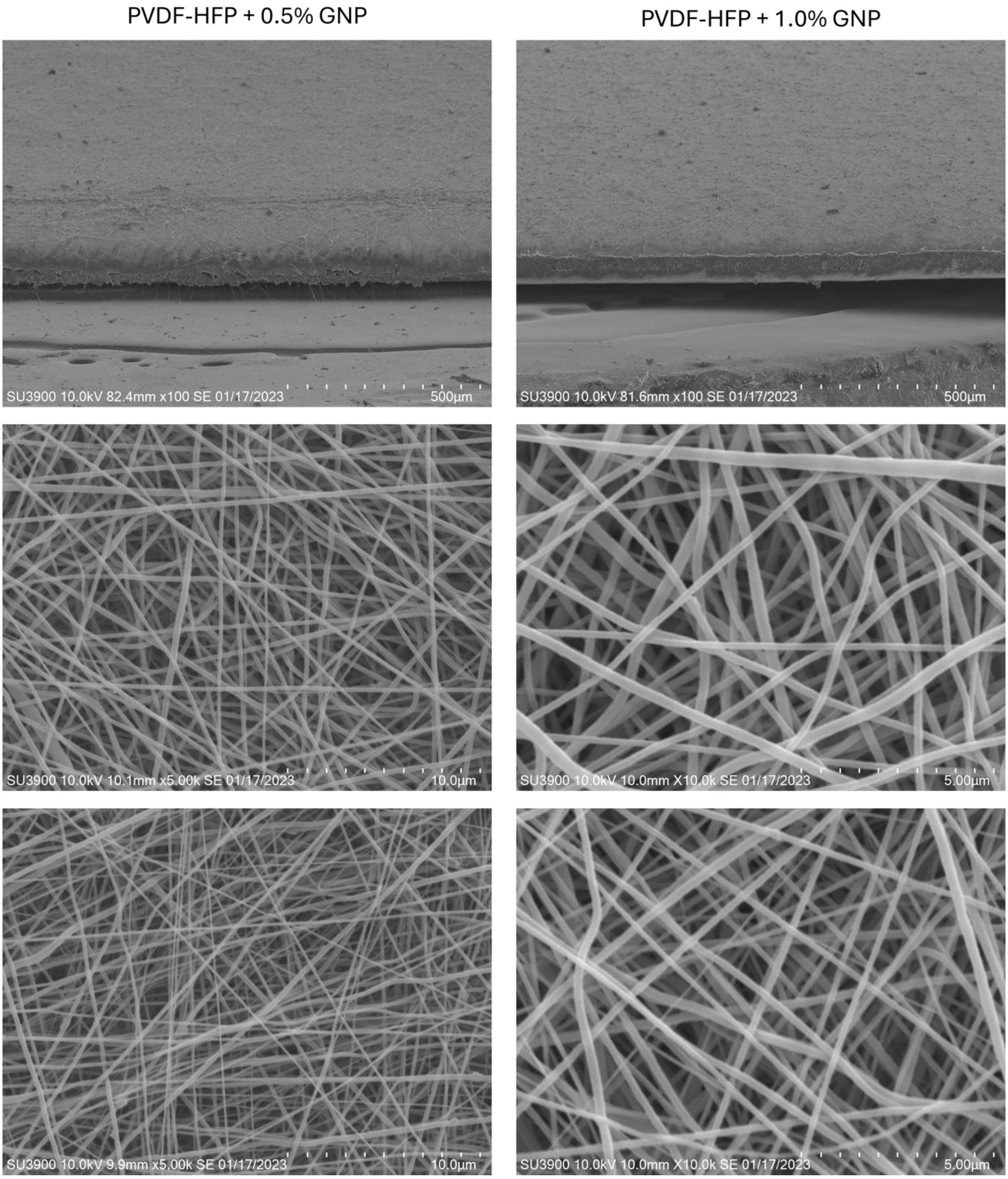

Figure S4. SEM micrographs of graphene nanoplatelets (GNP)-doped PVDF-HFP electrospun membranes. Uncropped, unmodified SEM micrographs illustrating the morphology of PVDF-HFP fibers doped with 0.5 wt% and 1.0 wt% GO, deposited onto a nylon-6 support substrate.

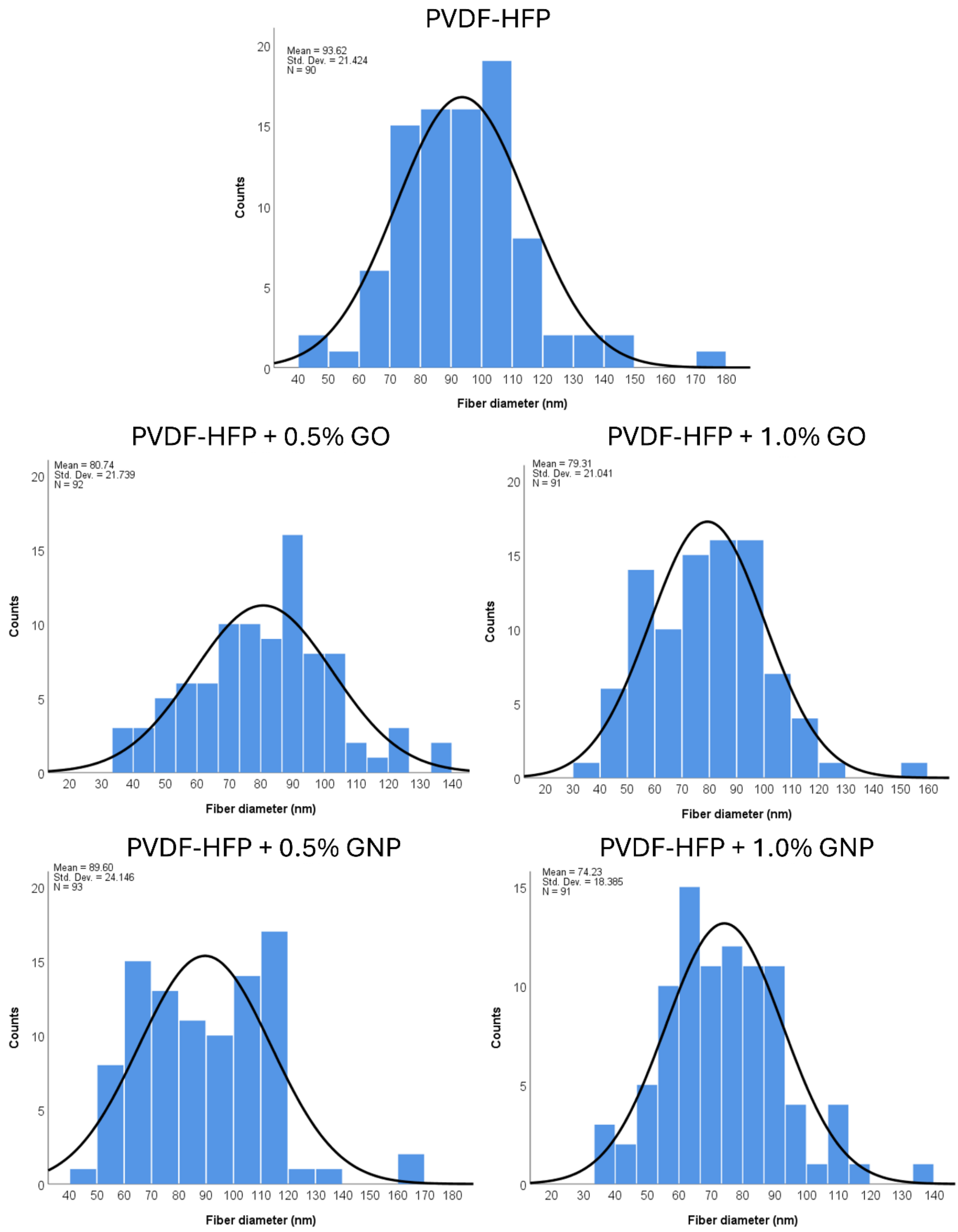

Figure S5. Fiber diameter distribution histograms of electrospun membranes. Histograms illustrating the fiber diameter distributions of pristine PVDF-HFP and doped with graphene oxide (GO) and graphene nanoplatelets (GNPs). Fiber diameter measurements were obtained using ImageJ 1.54f, and histograms were generated with IBM SPSS Statistics 25. N = Number of measured fiber widths is indicated by N.

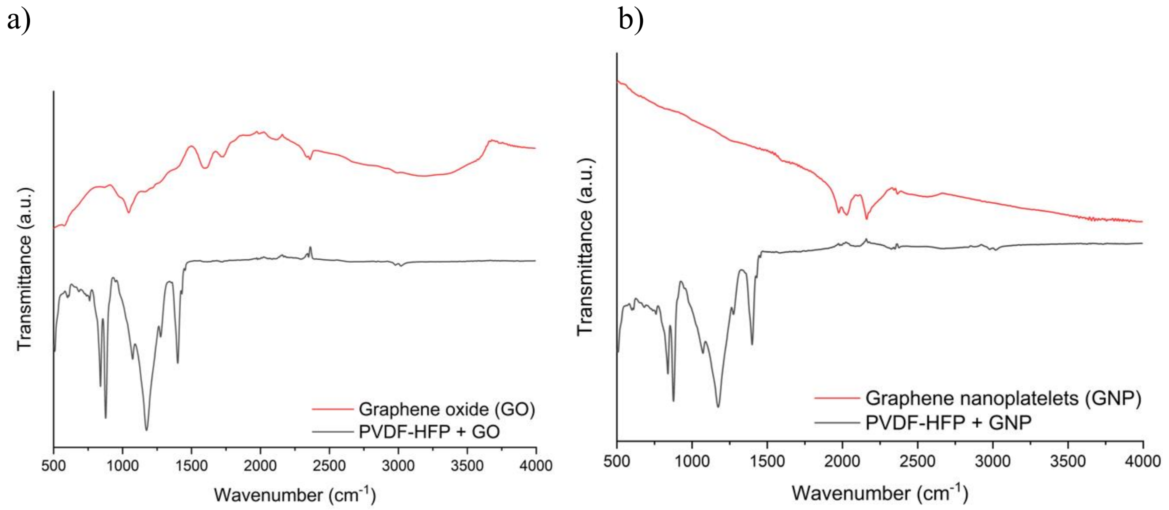

Figure S6. ATR-FTIR spectra of a) the graphene oxide (GO) powder and composite PVDF-HFP + GO electrospun membranes and of b) the graphene nanoplatelets (GNP) powder and composite PVDF-HFP + GNP electrospun membranes.

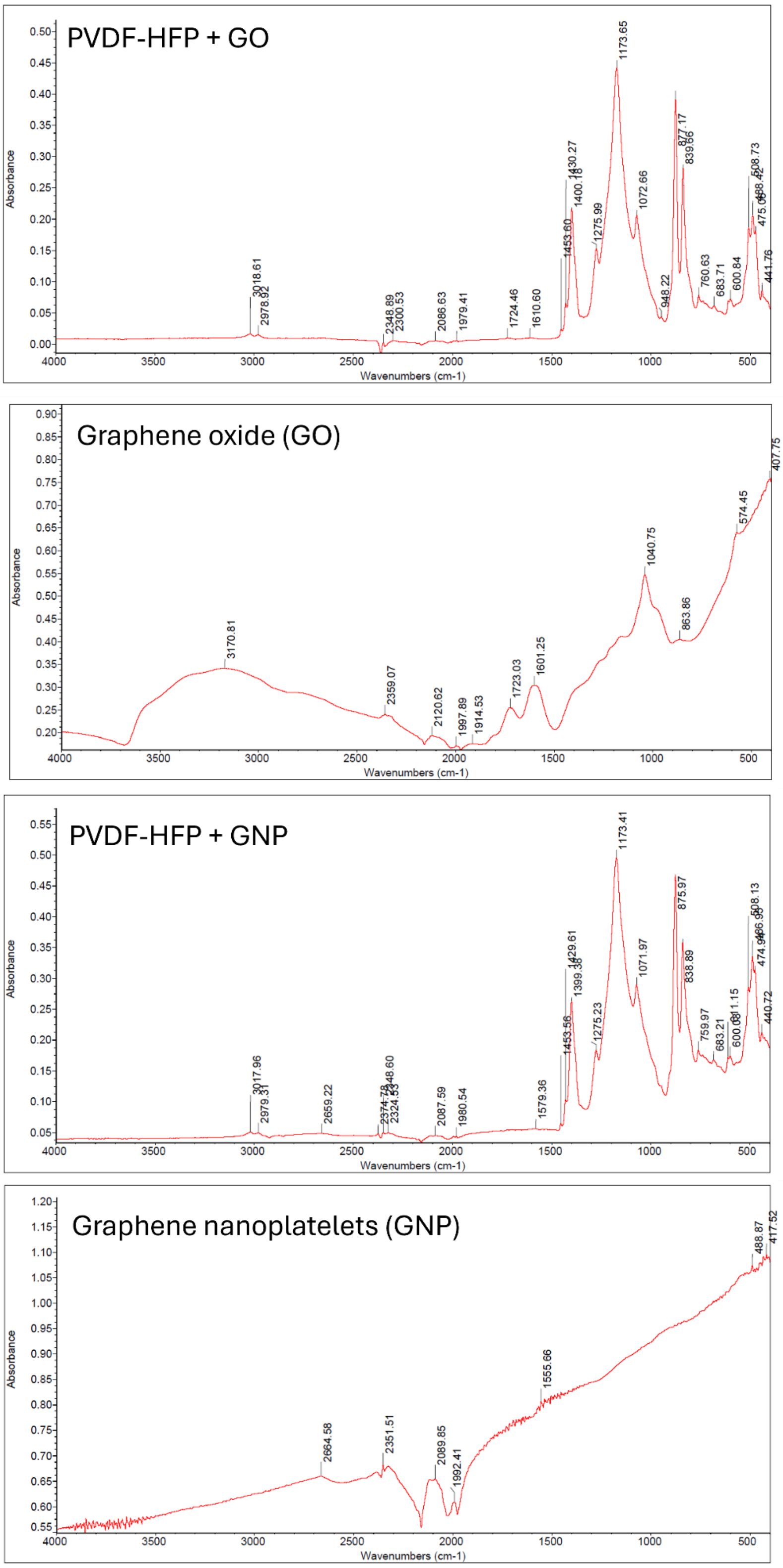

Figure S7. FTIR comparative analysis of absorbance spectra of the composite PVDF-HFP + GNP or GO vs the corresponding nano-powders. Peak analysis was conducted using the OMNIC™ spectroscopy analysis software (v. 9.2.86, Thermo Scientific).

Table S1. Comparative analysis of FTIR peak positions and functional group assignments for graphene nanoplatelets (GNP) and electrospun PVDF-HFP-GNP composite membranes. Peak analysis was conducted using the OMNIC™ spectroscopy analysis software.

Region (cm^{-1})	Graphene nanoplatelets (GNP)	PVDF-HFP + GNP	Shared Peak	Functional Group
**400-500**	418, 489	441, 475, 487	Yes	C-O bending or stretching (GNP), CF_2 group modes in PVDF
**500-600**	—	508, 600, 611	No	C-F stretching or C-O stretching (PVDF)
**600-700**	—	683	No	CF_2 wagging (PVDF)
**700-800**	—	760	No	CF_2 symmetric stretching (PVDF)
**800-900**	—	839, 876	No	C-H bending modes (PVDF backbone)
**900-1100**	—	1072	No	C-H stretching, bending in PVDF
**1100-1300**	—	1173, 1275	No	CF_2 stretching, CH_2 rocking in PVDF
**1300-1500**	—	1399, 1430, 1454	No	CH_2 scissoring, CF stretching modes (PVDF)
**1500-1700**	1556	1579	Yes	C=C stretching in GNP and aromatic/unsaturated groups in PVDF
**1900-2100**	1992, 2090	1981, 2088	Yes	C=O stretching, possible conjugated or unsaturated carbonyl groups
**2300-2400**	2352	2325, 2349, 2375	Yes	C≡C, CO_2 stretching (GNP)
**2600-2700**	2665	2659	Yes	C-H stretching vibrations
**2900-3200**	—	2979, 3018	No	CH stretching vibrations (PVDF)

Table S2. Comparative analysis of FTIR peak positions and functional group assignments for graphene oxide (GO) and electrospun PVDF-HFP-GNP composite membranes. Peak analysis was conducted using the OMNIC™ spectroscopy analysis software.

Region (cm^{-1})	Graphene oxide (GO)	PVDF-HFP + GO	Shared Peak	Functional Group
**400-500**	408	442, 475, 488	Yes	C-O bending or stretching, out-of-plane vibrations (related to epoxy groups in GO)
**500-600**	574	509, 601	No	C-F or C-O stretching, deformation in PVDF
**600-700**	—	684	No	CH_2 wagging (in PVDF) or CF_2 group stretching
**700-800**	—	761	No	CF_2 symmetric stretching in PVDF
**800-900**	864	840, 877	No	C-H bending (PVDF) or possible residual GO peaks
**900-1100**	1041	948, 1073	No	C-H bending, stretching (PVDF backbone) or OH groups in GO
**1100-1300**	—	1174, 1276	No	CF stretching, CH_2 rocking (PVDF)
**1300-1500**	—	1400, 1430, 1454	No	CH_2 scissoring, wagging (PVDF), bending
**1500-1700**	1601, 1723	1611, 1724	Yes	C=C stretching (graphene oxide, aromatic rings), carbonyl (C=O) stretching
**1900-2100**	1915, 1998, 2121	1979, 2087	Yes	C=O stretching or possible residual stretching modes (PVDF backbone)
**2300-2400**	2359	2301, 2349	No	C≡C, CO_2 stretching (GO)
**2900-3200**	3171	2979, 3019	No	CH stretching vibrations (in PVDF), residual OH stretching from GO

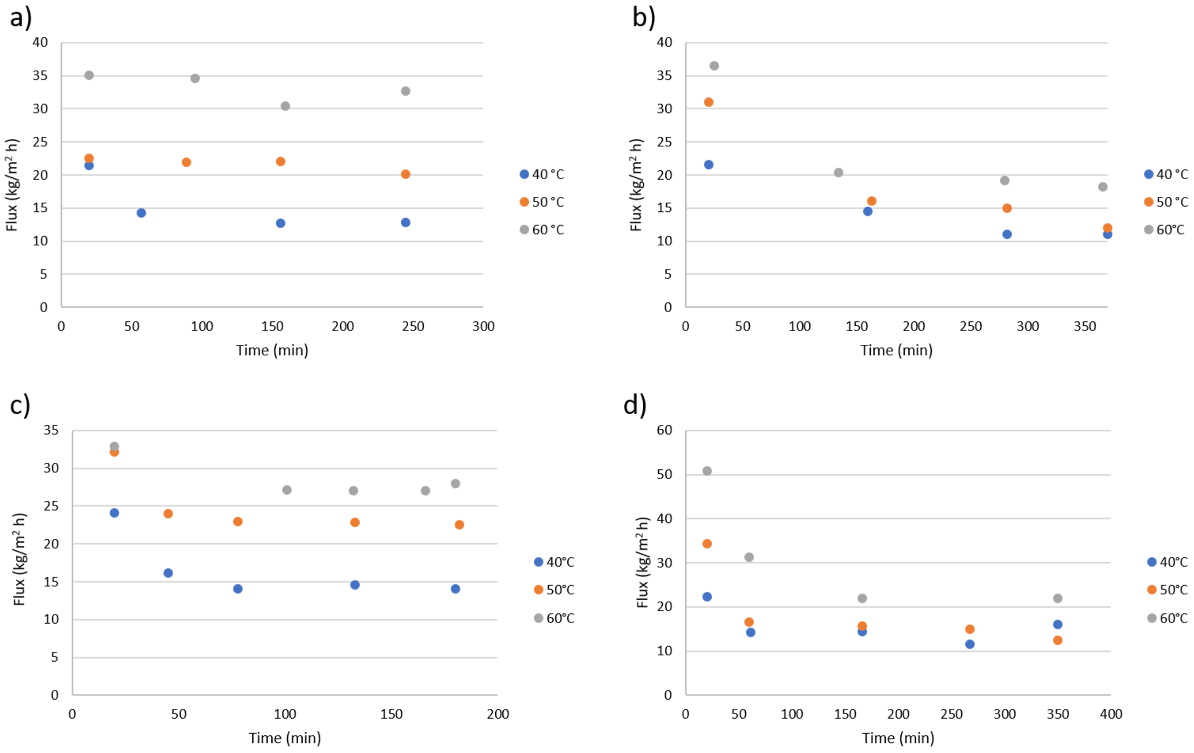

Figure S8. Trend of the permeate water fluxes and salt fluxes as a function of the feed temperature during VMD tests for the pristine PVDF-HFP membrane (a,b) and PVDF- HFP + 1% GNP membrane (c,d)
